# Relationships Between Osteocalcin, Glucose Metabolism, and Adiponectin in Obese Children: Is there Crosstalk Between Bone Tissue and Glucose Metabolism?

**DOI:** 10.4274/Jcrpe.831

**Published:** 2012-12-19

**Authors:** Nilay Abseyi, Zeynep Şıklar, Merih Berberoğlu, Bülent Hacıhamdioğlu, Şenay Savaş Erdeve, Gönül Öçal

**Affiliations:** 1 Ankara University School of Medicine, Department of Pediatiric Endocrinology, Ankara, Turkey

**Keywords:** obesity, osteocalcin, Adiponectin, hyperinsulinemia

## Abstract

**Objective:** Recently, scientific interest has focused on the association between osteocalcin, which originates from the skeletal system, and glucose metabolism. Although the association between lipid metabolism, adiponectin, and metabolic syndrome is well known, that between obesity, insulin resistance, and osteocalcin have not been clarified yet in children. The aim of this study was to assess the prevalence of insulin resistance in obese children and adolescents, as well as to investigate the effects of adiponectin and osteocalcin on the development of metabolic syndrome and insulin resistance.

**Methods:** A total of 150 obese nondiabetic children and adolescents, aged between 5 and 18 years, were included in the study. Serum adiponectin, osteocalcin and insulin levels were measured, and the association of the components of metabolic syndrome with adiponectin and osteocalcin levels was investigated. Insulin resistance was evaluated by Homeostasis model assessment insulin resistance (HOMA-IR).

**Results:** Metabolic syndrome was identified in 28% of the cases, all older than 10 years of age. No significant association was identified between insulin resistance, metabolic syndrome parameters, and osteocalcin levels. Adiponectin levels were significantly low in cases with metabolic syndrome, hyperinsulinemia, and in those with dyslipidemia. No significant association was found between adiponectin and osteocalcin levels.

**Conclusions:** We failed to show the effect of osteocalcin on insulin resistance in obese children and adolescents. This finding may be due to absence of hypergycemic blood glucose levels in our cases.

**Conflict of interest:**None declared.

## INTRODUCTION

Adipose tissue, which acts as an endocrine tissue, plays an important role in the regulation of insulin sensitivity both in the adipocytes and in other tissues (1). Studies have emphasized the fact that adiponectin, which is secreted from the adipose tissue and is known to increase insulin sensitivity, is found in remarkably low concentrations in patients with metabolic syndrome. It has been shown that adiponectin in vivo improves insulin resistance in many tissues (2).

In recent years, it has been reported that the skeletal system also plays a role in the regulation of energy and glucose metabolism (3). The presumed protective effect of obesity on osteoporosis has urged researchers to think about the presence of hormones that affect both the bones and the energy metabolism. The first rodent studies showed that osteocalcin, which is secreted from osteoblasts, increased insulin secretion as well as sensitivity to insulin. It has been shown in experimental animals that recombinant osteocalcin administration increases insulin sensitivity and adiponectin levels (4,5). Lee et al (5) demonstrated that osteocalcin is involved in glucose metabolism by increasing insulin secretion and cell proliferation in pancreatic β-cells and by upregulating the expression of the adiponectin gene in adipocytes, thus improving insulin sensitivity. Studies in adults and children, although few in number, have shown an association between low osteocalcin levels and insulin resistance (6,7,8). In addition, it is not known if osteocalcin has a role in improved insulin sensitivity associated with plasma adiponectin levels. Recently, it has been reported that in adults, circulating osteocalcin levels are associated with improved glucose tolerance and insulin secretion and sensitivity, independent of the plasma adiponectin level (9).

The association between obesity, insulin resistance, and osteocalcin, as well as the interaction between osteocalcin and adiponectin, has not yet been clarified in children, and the number of studies conducted in children and adolescents are limited.The aims of our study were as follows: 1) to assess the prevalence of insulin resistance in obese children and adolescents and to determine whether or not there is an association between insulin resistance and osteocalcin levels; 2) to identify whether or not there is a difference between patients with and without metabolic syndrome with respect to osteocalcin levels; 3) to determine the association between osteocalcin and the other components of metabolic syndrome (dyslipidemia, hypertension); 4) to investigate the relationships between osteocalcin levels, insulin resistance, and adiponectin levels.

## METHODS

The study was conducted with the participation of children and adolescents between 5 and 18 years of age admitted to our Pediatric Endocrinology Clinic. Inclusion criteria were as follows: 1. Being obese; 2. Absence of any other concomitant systemic disease (diabetes mellitus, chronic kidney disease, thyroid diseases, metabolic bone disease, hepatic disease, congenital heart disease); 3. Not receiving any medication with drugs known to effect glucose and bone metabolism such as metformin, glucocorticoids, bisphosphonates. Cases with an acute illness, such as infection, and with a history of recent trauma or fracture (<6 months) were excluded from the study.

Body weight was measured using a SECA® scale, with the subjects only lightly dressed. Height was measured barefoot using a fixed stadiometer with 1 mm-precision while the subject was standing with heels, buttocks, and shoulders against the wall. The measurements were evaluated based on age- and gender-specific reference values for Turkish children. Body mass index (BMI) was calculated using the following formula: body weight (kg) / height (m2). Children with a BMI between the 85th and 95th percentiles were considered overweight and those with a BMI > the 95th percentile were considered obese (10).

Waist circumference was measured midway between the 10th rib and the upper border of the iliac crest, while the subject was in upright position with a relaxed abdomen; hip circumference was measured. Measurements were evaluated according to the normal waist circumference values (11).

Blood pressure was measured in the morning while the subject was resting in a sitting position. Measurements were obtained from the right arm using a sphygmomanometer with a cuff of appropriate size (to cover 2/3 of the arm's length). The results were evaluated according to the Tumer's standards considering the normal values of age and gender (12). Cases with acanthosis nigricans detected on physical examination were recorded, and pubertal status was evaluated according to Marshall and Tanner (13,14).

Blood samples for laboratory analyses were obtained in the morning between 08.00 and 10.00 am after 12 hours of fasting. Fasting blood glucose, fasting insulin, lipid profile, aspartate aminotransferase (AST), and alanine aminotransferase (ALT) values were analyzed on the same day; whereas the serum was separated for the measurement of adiponectin and osteocalcin levels not later than an hour after the blood samples were obtained, and stored at -20°C. Glucoseand total cholesterol levels were enzymatically analyzedby hexokinase and oxidase methods, respectively. High-density lipoprotein (HDL)-cholesterol levels were analyzed by direct, non-immunological method. Triglyceride (TG) levels were calculated by the Friedewald equation if TG levels were <400 mg/dL or by homogenous enzymatic method if TG levels were >400 mg/dL using an automated analyzer (Roche®, Germany). Fasting insulin levels were detected by radioimmunoassay (RIA) method.

Osteocalcin levels were analyzed by electrochemiluminescence (ECL) method. Adiponectin levels were analyzed by enzyme-linked immunosorbent assay (ELISA) method using human adiponectin ELISA kits (Biovendor®).

After obtaining the results, insulin resistance was evaluated using fasting insulin levels and the homeostasis model assessment insulin resistance (HOMA-IR) index. Prepubertal girls with a HOMA-IR >2.22, prepubertal boys with a HOMA-IR >2.67, pubertal girls with a HOMA-IR >3.82 and pubertal boys with a HOMA-IR >5.22 were considered insulin resistant (15).

The diagnosis of metabolic syndrome was based on the International Diabetes Federation (IDF) criteria (16).

This study has been approved by local ethics committee. Informed consent was obtained by parents and, when appropriate, by children. 

## STATISTICAL ANALYSIS

Statistical analysis was carried out in the Department of Biostatistics at Ankara University Faculty of Medicine using the Statistical Package for the Social Sciences for Windows package program (version 15.0; SPSS Inc., Chicago, IL, USA). Descriptive statistics were expressed as means ± standard deviations (SD) or medians (min-max) for continuous variables, and as number of cases (n) and percentages (%) for nominal variables. A chi-square test was used for intergroup comparison of percentages. Multivariate logistic regression was used for significant univariate variables. The Odds ratio (OR) was given together with its 95% confidence interval (CI) for independent risk factors. A t-test was used for intergroup comparison of means when the variables were normally distributed, and the Mann-Whitney U test when the variables were not normally distributed.

A p-value <0.05 was considered statistically significant.

## RESULTS

The study included 150 obese (BMI > the 95th percentile) cases (54.7% boys and 45.3% girls) aged between 5 and 18 years. Mean age was 12.1±3.0 years (range, 5-17.4 years). The cases were grouped as prepubertal and pubertal (20.7% of the cases were prepubertal and 79.3% - pubertal). Mean age, body weight, height, BMI, and relative body mass index (RBMI) of the study group are given in Table 1.

Mean values for biochemical parameters [fasting blood glucose, fasting insulin, HOMA-IR, glucose/insulin, TG, low-density lipoprotein (LDL), HDL, very LDL and total cholesterol, calcium, phosphorus, alkaline phosphatase (ALP), AST, and ALT)] are shown in Table 2. 

Metabolic syndrome was found in 28% of the cases (all aged >10 years) according to the IDF criteria. The distribution of metabolic syndrome components (insulin resistance, hypertension, high TG, low HDL-cholesterol) were identified (Table 3). Hypertension was more common among boys (23% in boys vs. 9% in girls, p=0.019); however, no significant association was found between gender and the other components of metabolic syndrome (p<0.05).

Evaluation of osteocalcin and adiponectin levels by sex, age, and other characteristics of the cases is given in Table 4. An inverse association was found between age and osteocalcin levels (r=0.5, p=0.006). Osteocalcin levels were significantly lower in girls (p=0.00). No significant difference was found between prepubertal and pubertal cases with respect to mean osteocalcin levels (p= 0.617). There was also no significant association between high HOMA-IR values and mean osteocalcin levels. Osteocalcin levels were comparable in cases with and without hyperinsulinemia (p=0.26). Correlation analyses between osteocalcin levels and fasting insulin, fasting blood glucose, HOMA-IR, glucose/insulin, adiponectin, BMI-SD score (SDS), TG, LDL, HDL, and total cholesterol revealed no significant association. No significant association was found between osteocalcin levels and the presence of metabolic syndrome (p=0.129).

There was no significant difference between boys and girls with respect to adiponectin levels (p=0.247). Adiponectin levels were significantly higher in prepubertal cases as compared to pubertal cases (p=0.009). Adiponectin levels were lower in cases with hyperinsulinemia and high HOMA-IR. An inverse association was found between fasting insulin and adiponectin levels (r=0.366, p=0.013). Concerning the association between the presence of dyslipidemia (high TG or low HDL or both) and adiponectin levels, it was found that the median adiponectin level was 8.3 μg/mL (range, 3.1-16.9 μg/mL) in cases with dyslipidemia, while it was 10.1 μg/mL (range, 2.8-57.9 μg/mL) in those without dyslipidemia. The difference was statistically significant (p=0.013). The median adiponectin level was significantly lower in cases with low HDL-cholesterol level as compared to those with normal HDL-cholesterol level (p=0.03). Moreover, an inverse association was found between TG and adiponectin levels (r=0.255, p=0.002).

Comparison of the cases with and without metabolic syndrome with respect to adiponectin level revealed that adiponectin level was significantly lower in those with metabolic syndrome (p=0.00).

Correlation analysis demonstrated no significant association between adiponectin and osteocalcin levels (r=0.11, p=0.66).

## DISCUSSION

The association between the skeletal system and insulin resistance has started to attract the attention of researchers in recent years. Today, the skeletal system is considered as a member of the global energy mechanism. Osteoblasts secrete osteocalcin, which is the major non-collagen protein found in the extracellular matrix of bone. Since osteocalcin is produced only by osteoblasts, it is one of the factors that directly reflects bone metabolism (17).

The first studies demonstrating the association between glucose metabolism and osteocalcin in humans were conducted in type 2 diabetic adults. In 1998, Rosato et al (18) reported that osteocalcin levels were lower in subjects with type 2 diabetes than in healthy people. Another study analyzing serum osteocalcin levels in patients with type 2 diabetes showed that serum osteocalcin levels were lower in those with poor metabolic control as compared to those with good metabolic control and to the healthy control group (19). Osteocalcin may have different effects in diabetic and euglycemic cases. It is known that osteoblasts express glucose transporters GLUT1 and GLUT3. It has been shown that chronic hyperglycemia leads to an increase in ALP activity and to a decrease in osteocalcin secretion in osteoblasts (20).

In this present study on obese children and adolescents, no significant association between osteocalcin levels and the presence of metabolic syndrome was found. We were not able to detect the regulatory effect of osteocalcin on glucose metabolism. The absence of hyperglycemia in our cases might be one of the factors responsible for this finding. Interest in osteocalcin has increased following studies evaluating the adipose tissue in osteocalcin-deficient homozygous mice produced by ablation of the osteocalcin genes (5). Moreover, it was shown that blood glucose levels were higher and insulin levels lower in osteocalcin-deficient homozygous mice; the number and size of their pancreatic beta-cells were decreased. Osteocalcin-deficient homozygous mice also developed insulin resistance and glucose intolerance. Different from the effects of glucose on osteoblasts, the effects of osteocalcin on beta-cells have been shown. Particularly, the carboxylated form of osteocalcin was said to have a regulatory effect on insulin secretion (4,5). Carboxylated osteocalcin is involved in bone mineralization. The uncarboxylated form is not involved in mineralization but passes into the circulation.

It can be speculated that another reason for our failure to show the association between osteocalcin and insulin resistance in the present study might be the fact that we did not separately measure the levels of carboxylated and uncarboxylated osteocalcin. However, it has been demonstrated that both uncarbocxylated osteocalcin and serum osteocalcin change in a similar way with glucose loading (21,22). Hwang et al (21) evaluated the association between glucose metabolism and the plasma concentrations of uncarboxylated and carboxylated osteocalcin in 199 men and showed that HOMA-IR values were lower in cases with high osteocalcin levels. The authors reported that both carboxylated and uncarboxylated forms of osteocalcin improved the glucose tolerance (21).

In the present study, osteocalcin levels were found to be significantly lower in girls, and it was shown that pubertal stage did not affect osteocalcin levels. Reinehr et al (23) investigated the association between osteocalcin levels, puberty, and gender in obese children and reported significantly higher osteocalcin levels in boys. Similar to the present study, no association was established between pubertal stage and osteocalcin levels in that particular study.

In the present study, we found that osteocalcin levels decreased with increasing age. This finding is expected. Zhou et al (24) conducted a study in adults and showed that osteocalcin levels proportionally decreased as the age increased.

The evaluation of obese participants revealed no significant association between insulin resistance and osteocalcin levels in our study. There are conflicting results in children on this issue. Reinehr et al (23) found lower osteocalcin levels in obese children in their prospective study performed on 60 obese and 19 normal-weight children. In that particular study, a significant negative association was established between osteocalcin levels and insulin resistance. Two other studies showed low osteocalcin levels in cases with insulin resistance; in one of these studies, no correlation was reported between insulin resistance and osteocalcin levels (24,25,26). Rochefort et al (17), in their study involving 27 prepubertal obese children who had undergone training in physical activity with untrained children, found that in trained obese children, in addition to their increased energy metabolism and increased bone mineral density osteocalcin levels were increased and insulin levels were decreased. However, no significant correlation was found between adiponectin and osteocalcin levels. Comparing trained and untrained obese children, a correlation was found between insulin resistance and osteocalcin in trained obese children; such an association was not found in the untrained group. In addition to the association of osteocalcin with insulin resistance, its association with the other components of metabolic syndrome has been investigated as well. Im et al (27) found an association between osteocalcin levels and glucose metabolism; however, no significant association was found between osteocalcin and TG and HDL-cholesterol levels. No such association could be found in other studies performed on children (17,23). In the present study, we did not find any significant difference between patients with and without dyslipidemia with respect to osteocalcin levels.

In one study (5), recombinant osteocalcin was injected to mice fed with a high-fat diet and partially restored insulin sensitivity and glucose tolerance were found in mice receiving recombinant osteocalcin. Moreover, the authors emphasized the energy expenditure-increasing effect of osteocalcin by detecting additional mitochondria in the skeletal muscle of mice treated with recombinant osteocalcin. Additionally, the protective effect of recombinant osteocalcin against hepatic steatosis was shown in mice receiving recombinant osteocalcin. Osteocalcin metabolism in humans can be different and more complicated from that reported in animal studies.

Previous studies have stressed the fact that the levels of adiponectin, which is secreted from the adipose tissue and is known to increase insulin sensitivity, are remarkably low in cases with metabolic syndrome. It was reported that adiponectin levels were negatively associated with insulin and HOMA-IR values (28,29,30,31). In agreement with these reports, we also found an inverse association between adiponectin and fasting insulin levels, and an inverse relationship between adiponectin and TG levels. In addition, adiponectin levels were significantly higher in obese children who were prepubertal. It has been well documented that adiponectin is positively associated with HDL-cholesterol, whereas it is negatively associated with TG; that obese adolescents have lower adiponectin levels and a more atherogenic lipoprotein profile; and that adiponectin levels tend to be higher in the prepubertal and early pubertal stages as compared to the late pubertal stage (28,29).

In the present study, no correlation was found between osteocalcin and adiponectin levels. In a rodent study, blood glucose levels were found to be high and blood insulin levels low in osteocalcin-deficient homozygous mice. In order to detect whether or not osteocalcin exerted its effect through adiponectin, adiponectin- and osteocalcin-deficient heterozygous mice were produced. In these mice, normal insulin release and blood insulin levels, but decreased adiponectin levels and insulin sensitivity were shown. It is suggested that osteocalcin may exert its effect, even if just partially, through adiponectin (5). However, another study failed to show such an association between adiponectin and osteocalcin (23). In a recent study on adult subjects, it was reported that although the circulating osteocalcin level was associated with improved glucose tolerance and insulin secretion, this was independent of the plasma adiponectin level (9). We also thought that adiponectin and osteocalcin might have exerted their effects on insulin resistance through different mechanisms.

In conclusion, osteoblasts and adipocytes, the association between which has begun to attract attention in recent years, are said to be the partners of energy metabolism. In the present study, we failed to show the effect of osteocalcin, which has been introduced as the first energy-regulating factor of bone origin, on insulin resistance in obese children and adolescents. Blood glucose levels of our cases being not at hyperglycemic levels, might explain the absence of a significant association.

The present study showed the association between adiponectin and insulin resistance and metabolic syndrome components as previously described. No correlation was found between adiponectin and osteocalcin in obese children and adolescents in our study, none of which had diabetes or abnormal glucose tolerance. Thus, our subjects were free of the effect of glucose overload or hyperglycemia on osteocalcin secretion. Future investigations may help explain the relationship between glucose metabolism and bone tissue more clearly.

## Figures and Tables

**Table 1:**
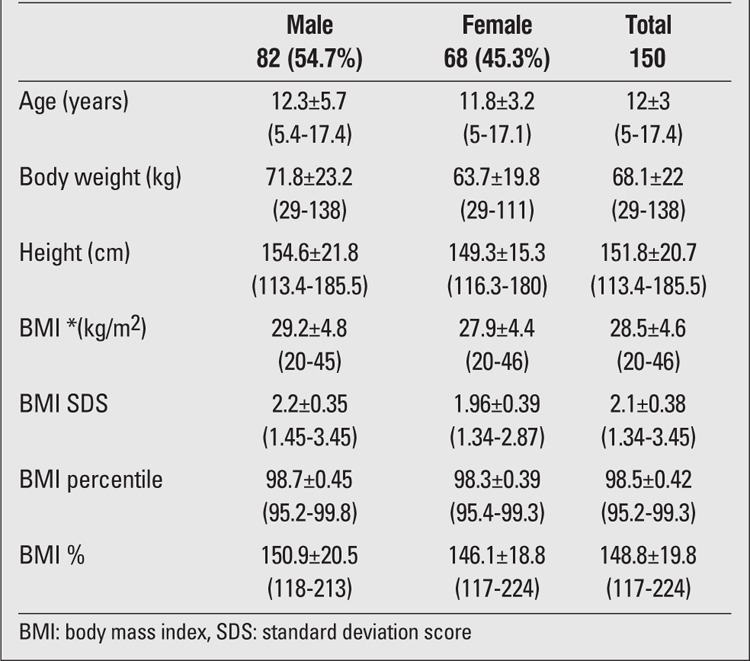
Characteristics of patients

**Table 2:**
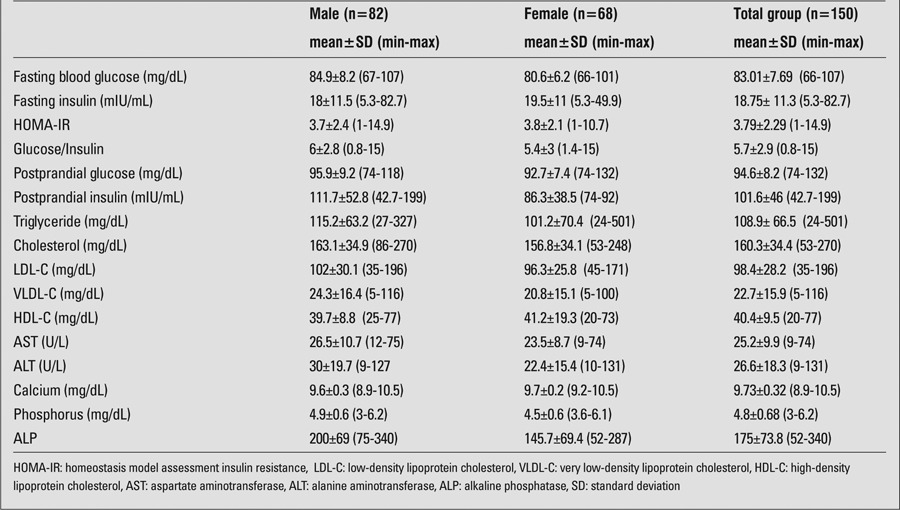
Laboratory values of the patients

**Table 3:**
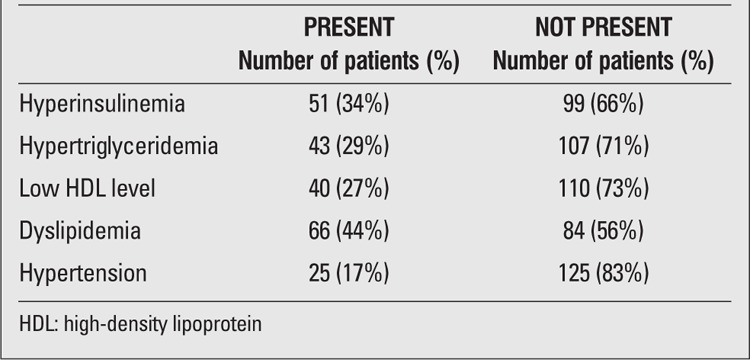
Results on the metabolic parameters of the patients

**Table 4:**
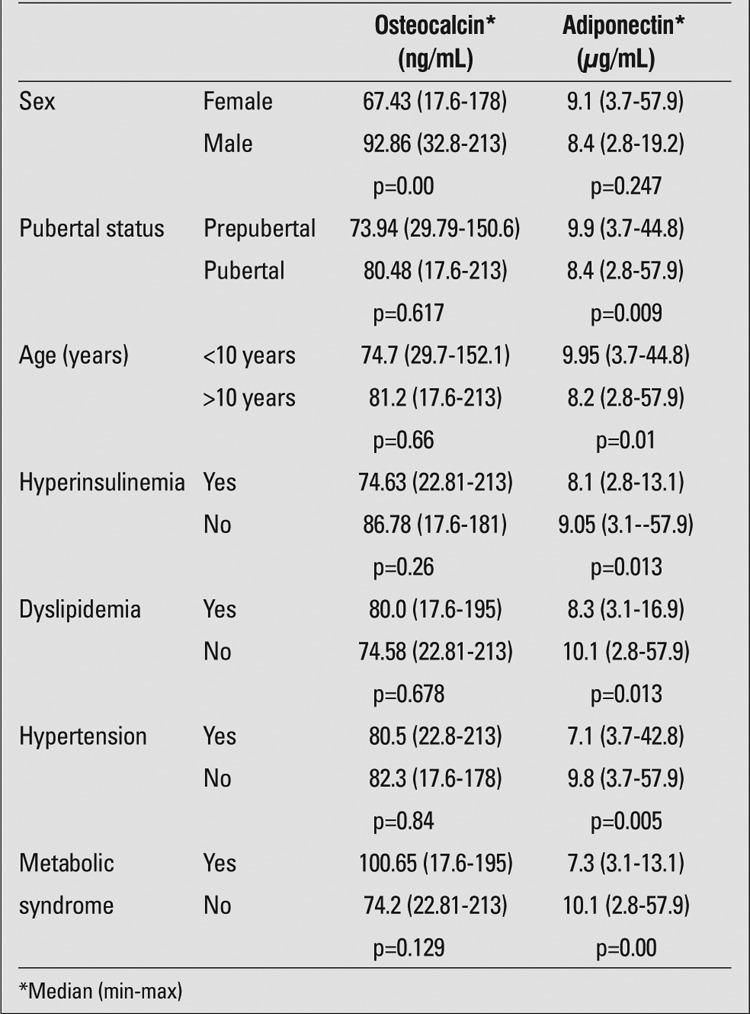
Patient characteristics according to osteocalcin andadiponectin levels
